# Atypical diabetic foot ulcer turning into squamous cell carcinoma

**DOI:** 10.1093/jscr/rjaf818

**Published:** 2025-10-16

**Authors:** Magdi S Alzir, Hani Mohammed Badahdah, Abdulelah K Alqawlaq

**Affiliations:** Orthopedics and Spine Surgery, International Medical Center, Hail Street, Al-Ruwais, 2172, Jeddah 21451, Saudi Arabia; Podiatric Foot & Ankle Surgery, International Medical Center, Hail Street, Al-Ruwais, 2172, Jeddah 21451, Saudi Arabia; College of Medicine and Surgery, Batterjee Medical College, Prince Abdullah Al‑Faisal Street, North Obhur, 6231, Jeddah 21442, Saudi Arabia

**Keywords:** diabetic foot ulcer, cutaneous squamous cell carcinoma, retrocalcaneal region, Marjolin’s ulcer, multidisciplinary management

## Abstract

Cutaneous squamous cell carcinoma (cSCC) may rarely arise in chronic diabetic foot ulcers, particularly at unusual sites. We report an 86-year-old man with a chronic retrocalcaneal ulcer refractory to standard care. Vascular imaging showed peripheral arterial disease, and biopsy confirmed well-differentiated cSCC (AJCC 8th edition: pT2Nx), measuring 2.1 × 1.5 cm with 8 mm invasion depth. Staging positron emission tomography/computed tomography and magnetic resonance imaging showed no evidence of metastasis. The patient underwent vascular optimization, wide local excision with clear margins, and local advancement flap coverage. At 4 months, there was no recurrence or metastasis, and wound healing was durable. To the best of our knowledge, this is the first reported case in Saudi Arabia of cSCC developing from a diabetic foot ulcer in the retrocalcaneal region. This case underlines the importance of early biopsy and multidisciplinary evaluation in atypical, non-healing ulcers to avoid delayed diagnosis and ensure optimal patient outcomes.

## Introduction

Cutaneous squamous cell carcinoma (cSCC) is the second most common non-melanoma skin cancer worldwide, representing 20%–50% of skin cancers [1, 2]. It usually arises from superficial epidermis, especially on sun-exposed skin or skin injury, or under chronic irritation [3, 4]. It occurs in middle-aged group and elderly adults, with a male predominance [1, 2]. Although most of the cases are managed effectively through surgical excision, a subset of cSCCs may exhibit local recurrence, metastasis, and in rare cases, lead to death [3].

In diabetic patients, neuropathy, peripheral arterial disease, and chronic ulcers create a microenvironment of inflammation that may lead to malignant transformation [4–7, 9]. One underrecognized risk factor is chronic non-healing ulcers, particularly in patients with diabetes mellitus. Chronic diabetic foot ulcers, in particular, have been implicated in rare cases of cSCC, often presenting as recurrent or non-resolving wounds with atypical features such as excessive granulation tissue, exophytic growth, or ulceration with raised, indurated edges [10, 11].

While cases of cSCC arising in diabetic foot ulcers have been described sporadically, they are uncommon and frequently undiagnosed, particularly in early stages of the disease. When it occurs as a complication of chronic osteomyelitis sinuses or longstanding ulcers of the plantar area, the diagnosis may be delayed, resulting in local invasion or limb amputation [12].

The aim of this study is to highlight the potential for malignant transformation of chronic diabetic foot ulcers into cSCC, a rare but serious complication that is often overlooked. By presenting a unique case of cSCC arising in the retrocalcaneal region of a chronic diabetic ulcer, this report underscores the importance of maintaining clinical suspicion, performing timely biopsies, and adopting a multidisciplinary approach in the evaluation and management of non-healing wounds in diabetic patients.

## Case report

An 86-year-old male patient with multiple comorbidities (DM, dyslipidemia, hypertension) presented with a history of a left leg ulcer for one year. The site of ulcer is in the retrocalcaneal area. The patient received local wound care and courses of antibiotics, but the ulcer failed to heal with no improvement. On initial presentation to our center, a thorough evaluation and examination were performed, and no history of trauma, fever, or signs and symptoms of systemic infection were identified.

**Figure 1:**
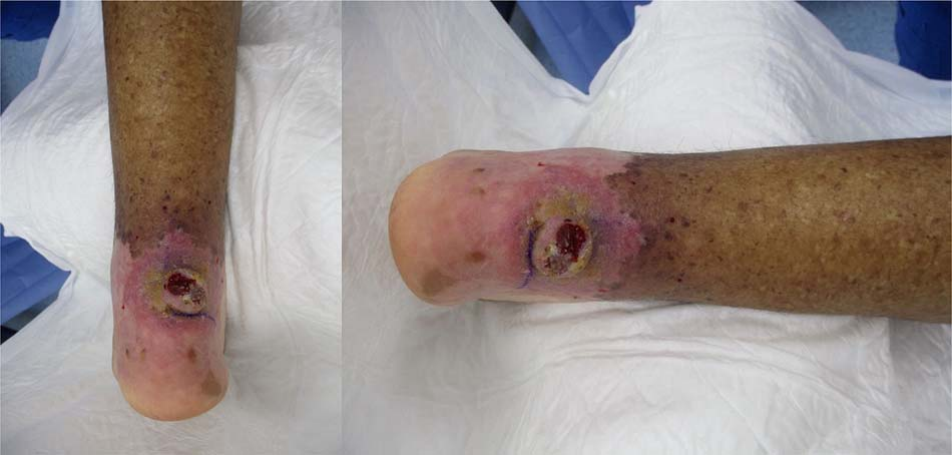
Chronic non-healing retrocalcaneal ulcer at the initial presentation.

On general examination, the patient appeared well and oriented without fever. Vascular examination revealed non-palpable pedal pulses. Neurological examination revealed: protective and epicritic sensation were absent on both feet. Orthopedic examination: limited range of motion at the left ankle, with muscle strength preserved. On local examination: A full-thickness 3 × 2 cm ulcer with irregular borders, fibrotic and granulation tissue base, and keratotic crusted edges was located in the retrocalcaneal area. There were no signs of erythema, purulent discharge, foul odor, or exposed bone. However, elevated skin temperature was noted around the ulcer (Fig. 1).

**Figure 2:**
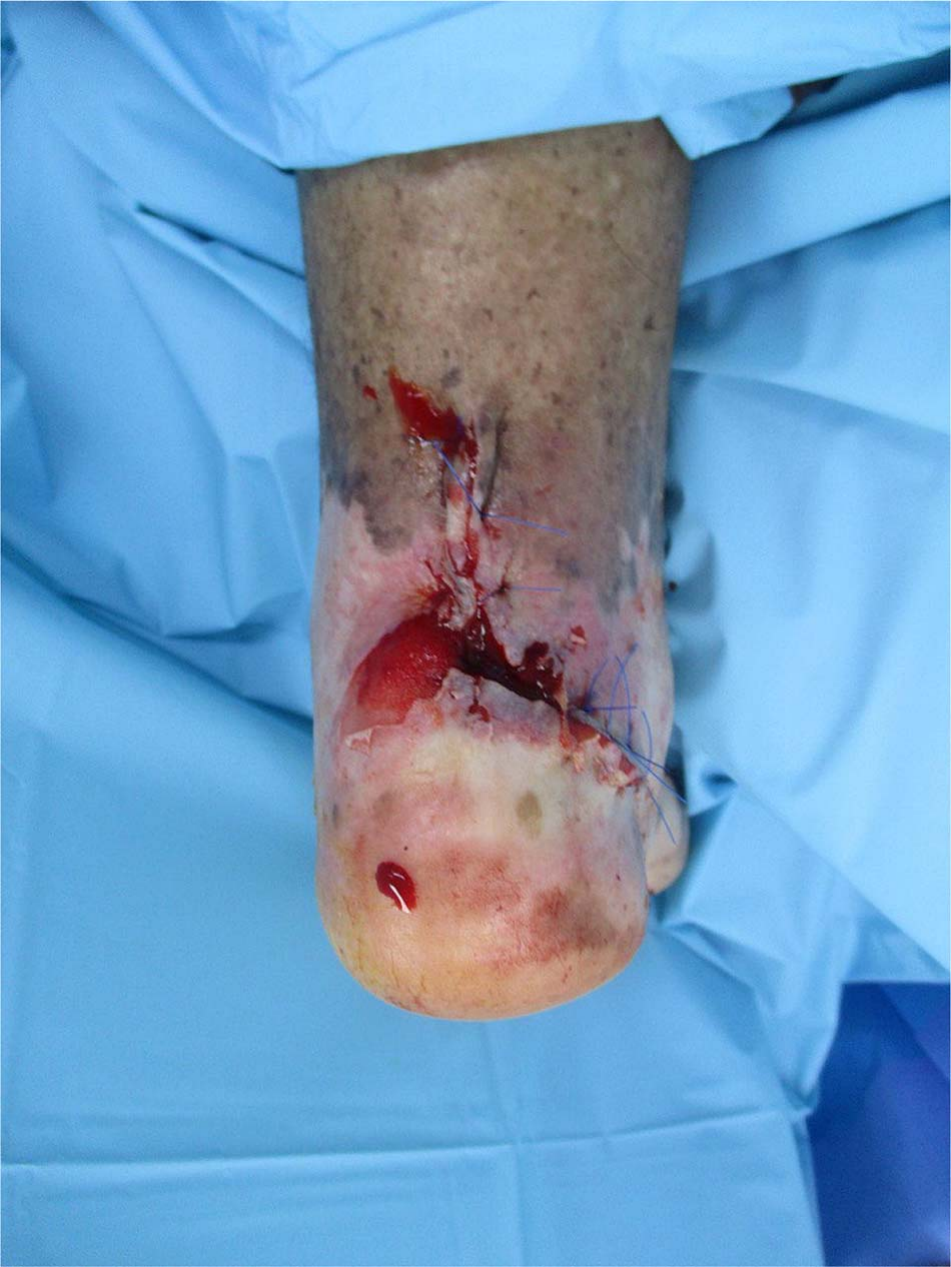
Intraoperative view showing wide local excision of the retrocalcaneal ulcer; histology confirmed well-differentiated SCC (pT2Nx) with 8 mm invasion depth; defect prepared for regional advancement flap coverage.

Vascular imaging showed atherosclerosis with femoral stenoses and posterior tibial artery occlusion; biopsy confirmed invasive cSCC (Fig. 2). An excisional biopsy during debridement of the ulcer edge was performed after vascular imaging and prior to definitive surgery, which confirmed invasive cutaneous SCC. Histopathology revealed a well-differentiated squamous cell carcinoma, pathological stage pT2Nx, and measuring 2.1 × 1.5 cm with a depth of invasion of 8 mm. The tumor reached anatomic level IV, invading the reticular dermis. No lymphovascular or perineural invasion was identified. The carcinoma was focally present at the lateral margin and 1 mm from the deep margin. Whole-body positron emission tomography/computed tomography and magnetic resonance imaging of the left foot showed only local inflammatory changes without evidence of regional or distant metastasis. Following multidisciplinary tumor board discussion, the patient underwent re-excision to achieve clear margins, which confirmed absence of residual carcinoma. Given the focally positive margin in the initial specimen, adjuvant radiotherapy was delivered post-operatively. Routine laboratory and radiographic investigations were unremarkable. At 4 months post-surgery and adjuvant radiotherapy, the patient remained disease-free.

## Discussion

Chronic, non-healing diabetic foot ulcers with irregular, indurated margins and keratotic/crusted edges should prompt early biopsy to exclude malignant transformation (Marjolin’s ulcer), although retrocalcaneal involvement is rare [1–3, 9]. Comparable malignant transformation has also been described in chronic heel ulcers [4]. In our patient, the chronicity and atypical edges despite standard care raised concern for malignancy, leading to excisional debridement and biopsy.

The differential diagnosis initially included neuropathic ulcer, chronic osteomyelitis, and venous ulcer. However, the ulcer’s atypical indurated margins, keratotic crust, and failure to respond to standard wound care raised suspicion for malignancy and prompted biopsy [3, 11].

Reported series of SCC arising in chronic ulcers describe higher risks of local recurrence and nodal spread compared with de novo cutaneous SCC [11], underscoring the need for wide local excision with clear margins, regional evaluation, and close surveillance. Reviews of malignant diabetic foot ulcers further emphasize the need for early recognition and aggressive surgical management [8]. In our patient, staging was consistent with AJCC 8th edition pT2Nx disease, with an 8 mm depth of invasion. Although margins were initially focally positive, re-excision and adjuvant radiotherapy ensured complete clearance and reduced the risk of recurrence. This highlights the importance of precise pathological staging, margin assessment, and multidisciplinary input in managing cSCC arising in diabetic foot ulcers, with systemic therapy options considered for advanced or unresectable disease [9]. In perfusion-limited diabetic limbs, a multidisciplinary approach including vascular optimization, oncologic resection, and reconstructive coverage can enable limb salvage with acceptable function, as achieved in this case.

**Figure 3:**
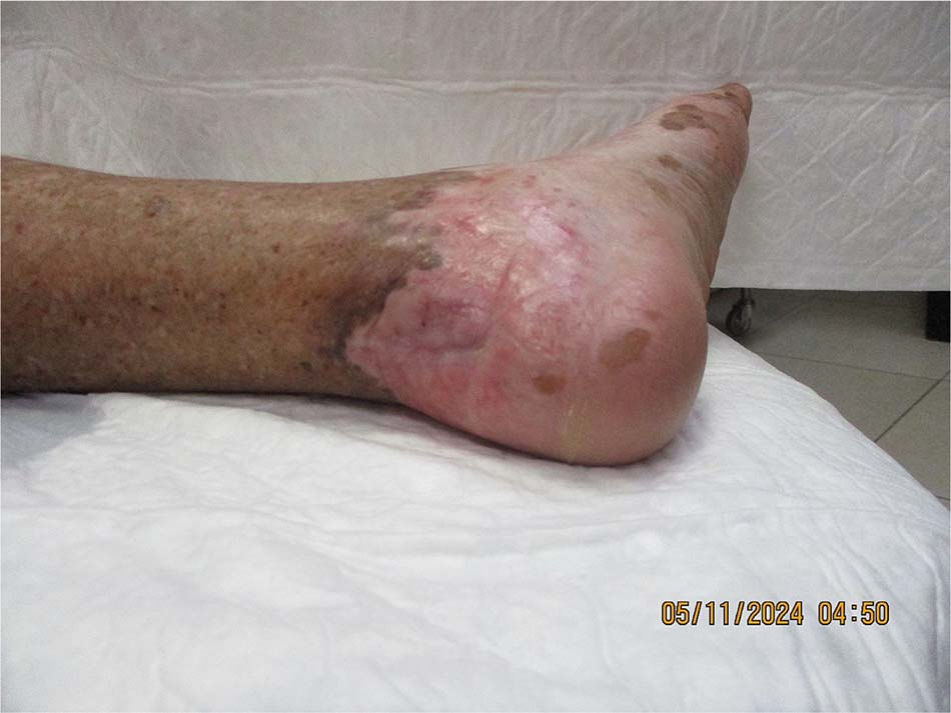
Completely healed retrocalcaneal region at 4 months post-surgery and adjuvant radiotherapy following regional advancement flap reconstruction, demonstrating durable coverage and limb preservation.

Peripheral arterial disease and diabetic neuropathy can mask early malignant change and delay presentation, as likely occurred in this case [5, 6, 10]. Similar malignant transformations have also been reported in chronic burn scars [10]. Interventions with the goal of improving perfusion of the vasculature, oncological evaluation for staging of malignancy, and surgical management with complete excision and reconstruction are required to achieve the best outcomes. Close medicosurgical collaboration achieved global management and, ultimately, curative limb treatment with limb salvage surgery [12]. In chronic ulcers such as SCC, complete excision with clear margins is crucial, as inadequate resection increases recurrence and metastasis risk [12]. In the present case, early identification, vascular optimization, and regional advancement flap reconstruction led to wound healing and functional limb preservation (Fig. 3). Reports of SCC in atypical anatomical sites underscore the importance of vigilance in unusual locations [12].

At 4 months of follow-up, the wound remained healed with no local recurrence or distant metastasis, and the patient was ambulatory with protective footwear.

## Conclusion

Atypical, non-healing diabetic foot ulcers warrant early biopsy to exclude malignancy. In perfusion-limited limbs, multidisciplinary management including vascular optimization, oncologic excision with clear margins, and appropriate reconstruction facilitates timely diagnosis, durable healing, and limb preservation.

## Data Availability

Not applicable.
